# Cefiderocol susceptibility of *Achromobacter* spp.: study of an accurately identified collection of 230 strains

**DOI:** 10.1186/s12941-024-00709-z

**Published:** 2024-06-17

**Authors:** Vincent Jean-Pierre, Pauline Sorlin, Alix Pantel, Raphaël Chiron, Jean-Philippe Lavigne, Katy Jeannot, Hélène Marchandin, Collaborative study group on antimicrobial resistance of Achromobacter spp., Marlène Amara, Lucile Cadot, Olivier Dauwalder, Nicolas Degand, Magalie Demar, Clarisse Dupin, Marie-Sarah Fangous, Claire Franczak, Fabien Garnier, Pascal Guiet, Jérôme Guinard, Cécile Hombrouck-Alet, Atika Kaoula, Patricia Mariani-Kurkdjian, Niels Nørskov-Lauritsen, Frédéric Schramm, Charlotte Tellini, Anthony Texier, Jérémie Violette, Nathalie Wilhelm

**Affiliations:** 1 HydroSciences Montpellier, Univ. Montpellier, CNRS, IRD, Service de Microbiologie et Hygiène Hospitalière, CHU de Nîmes, 34093 Montpellier, France; 2 VBIC, INSERM U1047, Univ. Montpellier, Service de Microbiologie et Hygiène Hospitalière, CHU de Nîmes, 30029 Nîmes Cedex 9, France; 3 HydroSciences Montpellier, Univ. Montpellier, CNRS, IRD, Centre de Ressources et de Compétences de la Mucoviscidose, CHU de Montpellier, 34093 Montpellier, France; 4 Laboratoire Associé Au Centre National de Référence de La Résistance Aux Antibiotiques, CHU de Besançon, 25000 Besançon, France

**Keywords:** *Achromobacter*, Opportunistic pathogen, Cystic fibrosis, Cefiderocol, Susceptibility

## Abstract

**Background:**

*Achromobacter* spp. are opportunistic pathogens, mostly infecting immunocompromised patients and patients with cystic fibrosis (CF) and considered as difficult-to-treat pathogens due to both intrinsic resistance and the possibility of acquired antimicrobial resistance. Species identification remains challenging leading to imprecise descriptions of resistance in each taxon. Cefiderocol is a broad-spectrum siderophore cephalosporin increasingly used in the management of *Achromobacter* infections for which susceptibility data remain scarce. We aimed to describe the susceptibility to cefiderocol of a collection of *Achromobacter* strains encompassing different species and isolation sources from CF or non-CF (NCF) patients.

**Methods:**

We studied 230 *Achromobacter* strains (67 from CF, 163 from NCF patients) identified by *nrdA* gene-based analysis, with available susceptibility data for piperacillin–tazobactam, meropenem and trimethoprim–sulfamethoxazole. Minimal inhibitory concentrations (MICs) of cefiderocol were determined using the broth microdilution reference method according to EUCAST guidelines.

**Results:**

Strains belonged to 15 species. *A. xylosoxidans* represented the main species (71.3%). MICs ranged from ≤ 0.015 to 16 mg/L with MIC_50/90_ of ≤ 0.015/0.5 mg/L overall and 0.125/2 mg/L against 27 (11.7%) meropenem-non-susceptible strains. Cefiderocol MICs were not related to CF/NCF origin or species although *A. xylosoxidans* MICs were statistically lower than those of other species considered as a whole. Considering the EUCAST non-species related breakpoint (2 mg/L), 228 strains (99.1%) were susceptible to cefiderocol. The two cefiderocol-resistant strains (*A. xylosoxidans* from CF patients) represented 3.7% of meropenem-non-susceptible strains and 12.5% of MDR strains.

**Conclusions:**

Cefiderocol exhibited excellent in vitro activity against a large collection of accurately identified *Achromobacter* strains, irrespective of species and origin.

**Supplementary Information:**

The online version contains supplementary material available at 10.1186/s12941-024-00709-z.

## Introduction

*Achromobacter* spp. are obligately aerobic, nonfermenting Gram-negative bacilli (GNB), belonging to the order Burkholderiales which are widely distributed in the environment (mostly soil and water) and also opportunistic pathogens in humans [1–3]. Accurate species identification is challenging, as both matrix-assisted laser desorption/ionization-time of flight mass spectrometry (MALDI-TOF MS) and 16S rRNA gene sequencing are inadequate to accurately distinguish species of the *Achromobacter* genus and often misidentify *Achromobacter* species as *Achromobacter xylosoxidans* [4]. Consequently, the true frequency of the various species of *Achromobacter* remains poorly defined leading to an imprecise description of specificities of each taxon. Unlike these conventional identification methods frequently used in former studies, multilocus sequence typing (MLST) and *nrdA* gene sequencing (765 bp) have proved to be highly discriminatory tools for species-level identification of *Achromobacter* strains [4, 5]. Studies based on these techniques identified *A. xylosoxidans* as the most frequent species recovered from clinical samples worldwide [4, 6] followed by *Achromobacter insuavis* in both cystic fibrosis (CF) [7, 8] and non-CF (NCF) patients [9, 10]. However, other species also infect humans and 20.6% of *Achromobacter* strains isolated from diverse non-respiratory samples of NCF patients in France belonged to *Achromobacter aegrifaciens*, *Achromobacter animicus*, *Achromobacter denitrificans*,* Achromobacter dolens*, *Achromobacter insolitus*, *Achromobacter marplatensis*,* Achromobacter mucicolens*, *Achromobacter spanius* and genogroup 9 [9], whereas 48.1% of *Achromobacter* spp. infections in CF patients in the United States involved *Achromobacter ruhlandii*, *Achromobacter dolens*, *Achromobacter insolitus* and *Achromobacter aegrifaciens* [4].

To date, minimal inhibitory concentration (MIC) and inhibition zone diameter (IZD) breakpoints are only edited by EUCAST (European committee on antimicrobial susceptibility testing) for the *A. xylosoxidans* species and for the three antibiotics piperacillin–tazobactam (TZP), meropenem (MEM) and trimethoprim–sulfamethoxazole (SXT) [11], reported as being the most effective in vitro against this species [12]. Indeed, *Achromobacter* spp. are intrinsically resistant to several antibiotics (e.g., most cephalosporins apart from ceftazidime, aztreonam, ertapenem and aminoglycosides), and are likely to acquire additional resistance, notably to TZP, MEM and SXT leading to the emergence of multidrug resistant (MDR) strains resulting in limited treatment options [13, 14]. None of the new β-lactam/β-lactamase inhibitor combinations (e.g., ceftolozane–tazobactam, ceftazidime–avibactam, imipenem–relebactam, meropenem–vaborbactam) appear to be therapeutic options of interest for managing infections caused by MDR *Achromobacter* strains [15], which explains the growing interest in new antibiotics with original mechanisms of action.

Cefiderocol is a new broad-spectrum antimicrobial drug approved by the U.S. Food and Drug Administration in 2019 and by the European medicines agency in 2020, and then available in France since January 2021 after a favourable opinion issued by the French National Authority for Health for the treatment of infections due to multiresistant aerobic GNB (including Enterobacterales and nonfermenting GNB) in adults with limited therapeutic options [16, 17]. Cefiderocol is an injectable siderophore cephalosporin conjugated with a catechol moiety on its side chain using a “Trojan horse” strategy [18]. The original cephalosporin structure provides stability against hydrolysis by nearly all β-lactamases including class B β-lactamases [15]. The catechol moiety enables cefiderocol to mimic natural siderophores by binding to ferric iron (Fe^3+^), and to cross the outer membrane through the active iron-transport systems of GNB. Once inside the bacterial periplasmic space, the cephalosporin core has a high affinity for penicillin-binding proteins (PBP), mainly PBP3, allowing cefiderocol to inhibit biosynthesis of the cell wall peptidoglycan, causing cell death [19].

Cefiderocol is increasingly used in the management of *Achromobacter* infections and already appears to be a promising therapeutic option [14, 20–29]. To the best of our knowledge, although the EUCAST has published susceptibility data on Enterobacterales and the nonfermenting GNB *Pseudomonas aeruginosa*, *Acinetobacter baumannii* and *Stenotrophomonas maltophilia*, to date there have been no studies describing the susceptibility of cefiderocol for *Achromobacter* spp. with reliable identification of the various species. Here, we evaluated susceptibility to cefiderocol on a collection of 230 *Achromobacter* strains encompassing different species accurately identified by *nrdA* gene sequence analysis and different isolation sources (NCF or CF) with the broth microdilution (BMD) reference method, and assessed MIC variability according to species and origin of strains.

## Materials and methods

### *Achromobacter* spp. collection and species identification

A total of 230 clinically-documented strains of *Achromobacter* spp. were selected, including 67 strains from the sputum of 67 CF patients (none of whom had received cefiderocol) and 163 strains from 163 NCF patients (Table 1). The strains were isolated between 2010 and 2023 during routine microbiological analysis of samples from patients attending (i) the CF centers (CRCM, Centre de Ressource et de Compétence de la Mucoviscidose) of the University Hospitals of Paris, Montpellier (France) or Aarhus (Denmark), (ii) one of the 6 French University Hospitals of Limoges, Lyon, Montpellier, Nîmes, Orléans and Strasbourg or one of the 14 French General Hospitals of Alès-Cévennes, Antibes-Juan les Pins, Blois, Bourgoin-Jallieu, Cahors, Cayenne, Mâcon, Montélimar, Quimper-Concarneau, Saint Brieuc, Saintes, Sens, Metz-Thionville, and Versailles for NCF patients (Additional file 1).

**Table 1:** Origin of the 230 *Achromobacter* sp. strains in this study

	Origin	Number of strains	Percentage of strains among CF/NCF (%)	Total percentage of strains (%)
CF (n = 67)	Sputum	67	100	29.1
NCF (n = 163)	Respiratory tract sample:	75	46	32.6
	Endobronchial aspirate	27	16.6	11.8
	Sputum	24	14.7	10.4
	Bronchoalveolar fluid lavage	18	11	7.8
	Distal airway secretions	6	3.7	2.6
	Blood culture	25	15.3	10.9
	Ear-nose-throat sample	14	8.6	6.1
	Skin and soft tissue biopsy	6	3.7	2.6
	Skin wound and pus	6	3.7	2.6
	Bone biopsy	6	3.7	2.6
	Implantable device	4	2.5	1.7
	Urine	3	1.9	1.3
	Rectum	2	1.2	0.9
	Eye	2	1.2	0.9
	Peritoneal fluid	2	1.2	0.9
	Ascites fluid	1	0.6	0.4
	Non specified	17	10.4	7.4

CF: strain(s) from patient(s) with cystic fibrosis; NCF: strain(s) from other patient(s) not suffering from cystic fibrosis

Most strains originated from the respiratory tract (100% of CF strains and 46% of NCF strains), followed by blood cultures (15.3% of NCF strains) and ear-nose-throat samples (8.6% of NCF strains) (Table 1). Other strains (30.1% of NCF strains) with known origin were isolated from skin wound and pus, biopsies, the digestive tract, implantable devices or eyes (Table 1).

Susceptibility data for TZP, MEM and SXT were available for the 230 strains based on the disk diffusion method using Bio-Rad disks (Bio-Rad Laboratories, Hercules, CA) on Difco™ Mueller–Hinton (MH) agar plates (Becton Dickinson, Pont-de-Claix, France). Among the 230 strains, most isolates were susceptible to TZP (90%), MEM (88.3%) and SXT (84.8%), applying the breakpoints of *A. xylosoxidans* to all *Achromobacter* species [11] (Additional file 1).

Species had been identified by *nrdA* gene sequence determination, analysis, and phylogeny [6]. Briefly, *nrdA* genes were amplified as previously described [4]. Taxonomic assignment was performed either using PubMLST database (https://pubmlst.org/organisms/achromobacter-spp) or after reconstructing a maximum-likelihood tree based on *nrdA* partial sequences (765 bp) and including all the type strains of *Achromobacter* species with validly published names and species with non-validly-published names, according to the list of prokaryotic names with standing in nomenclature (LPSN) (https://lpsn.dsmz.de/genus/achromobacter), as well as representative strains of *Achromobacter* genogroups available on PubMLST database [6]. All strains were stored frozen at − 80 °C in glycerol Trypticase-Soy broth.

### Antimicrobial susceptibility testing (AST) of cefiderocol with BMD reference method of *Achromobacter* spp.

Reference MIC values were determined by the National Reference Centre for Antibiotic Resistance (Besançon, France) by using an iron-depleted cation-adjusted Mueller–Hinton broth (ID-CAMHB) as described previously by Devoos et al*.* [30]. A commercial MH broth (Becton Dickinson, Pont-de-Claix, France) was processed twice with Chelex® 100 resin (Bio-Rad Laboratories, Hercules, CA) to remove iron and other cations in the medium (*i.e.*, calcium, magnesium and zinc). The iron-depleted broth was passed through a 0.22 µm filter to remove the resin and the final pH was adjusted to (7.2–7.4) using 0.1 M hydrochloric acid. Following this process, cations were added back to concentrations of calcium 20–25 mg/L, magnesium 10–12.5 mg/L, and zinc 0.5–1.0 mg/L [31]. The final concentration of iron was measured at < 0.03 mg/L by flame spectrometry (QUALIO, Besançon, France), according to quality standard ISO 11885. The BMD panels were incubated at 35 °C for 20 h in ambient air before MIC endpoints were read. If strong growth was not observed in the growth control well, the panels were incubated for a further 24 h. MICs were determined separately by two operators and confirmed by a third operator in the event of disagreement. Quality control using *Pseudomonas aeruginosa* strain CIP 76110 (= ATCC 27853) was included in each series of experiments to ensure the validity of the method, checking that the results were within the specified range (0.06 to 0.5 mg/L).

### Data analysis

The EUCAST 2023 pharmacokinetics and pharmacodynamics (PK/PD) breakpoint not related to a species for cefiderocol is 2 mg/L (susceptible strain: MIC ≤ 2 mg/L; resistant strain: MIC > 2 mg/L). MIC_50_ and MIC_90_ represent the MIC values at which the growth of ≥ 50% and ≥ 90% of the strains is inhibited, respectively.

All the statistical tests were performed using GraphPad Prism (GraphPad Software, La Jolla, CA). A two-tailed *p-value* < 0.05 was appointed statistically significant. A Kruskal–Wallis test was used to determine whether there was a significant relationship between the MICs of cefiderocol and the species in the *Achromobacter* genus. Wilcoxon tests were used to determine (i) whether there was a significant relationship between the MICs of cefiderocol of *A. xylosoxidans* and those of other species of the *Achromobacter* genus, and (ii) whether there was a significant relationship between the MICs of cefiderocol and the origin (CF or NCF) of the strains.

## Results

### Species diversity within the collection of *Achromobacter* strains studied

A high genetic diversity was observed among the collection with 62 alleles of the *nrdA* gene detected. The 230 strains studied were assigned to 15 species by *nrdA*-gene-based analysis including two potential new species (Additional file 1). Distribution of the 230 strains in the 15 species identified according to whether they were of CF or NCF origin is presented in Table 2. *A. xylosoxidans* was the most represented species (71.3% of strains, n = 164), in both CF and NCF groups (62.7% of CF strains, and 74.8% of NCF strains) followed by *A. insuavis* (7.4% of strains (n = 17), 13.4% of CF strains and 4.9% of NCF strains). Thirteen other species grouped less than nine strains including four species comprising a single strain (*A. kerstersii*, genogroup 19, genogroup 21 and genogroup 3). A higher diversity of species was noted for strains from NCF patients (n = 13) compared with strains from CF patients (n = 11). Most species were identified in both CF and NCF groups except for two species which were only identified in the CF group (genogroup 19 and genogroup 21), and four species only identified in the NCF group (*A. marplatensis*, *A. kerstersii*, new species 1, and genogroup 3) (Table 2).

**Table 2:** Cefiderocol susceptibility of the 230 *Achromobacter* sp. strains, according to species, origin (NCF or CF) and susceptibility to TZP, MEM and SXT

	n	NCF/CF	Range (mg/L)	MIC_50_^*^ (mg/L)	MIC_90_^*^ (mg/L)	Susceptibility^**^ (%)
All isolates	230	163/67	≤ 0.015 to 16	≤ 0.015	0.5	99.1
According to species						
*A. xylosoxidans*	164	122/42	≤ 0.015 to 16	≤ 0.015	0.5	98.8
*A. insuavis*	17	8/9	≤ 0.015 to 2	0.25	2	100
New species 1	8	8/0	≤ 0.015 to 0.06	0.06	0.06	100
*A. mucicolens*	8	6/2	0.03 to 0.25	0.125	0.25	100
*A. marplatensis*	6	6/0	0.06 to 0.125	0.125	0.125	100
*A. insolitus*	5	2/3	≤ 0.015 to 0.06	0.03	0.06	100
*A. ruhlandi*	5	3/2	≤ 0.015 to 0.5	≤ 0.015	0.5	100
*A. aegrifaciens*	4	1/3	≤ 0.015 to 0.25	0.06	0.25	100
*A. animicus*	4	3/1	≤ 0.015 to 0.06	0.03	0.06	100
New species 2	3	1/2	≤ 0.015 to 0.06	–	–	100
*A. dolens*	2	1/1	≤ 0.015	–	–	100
*A. kerstersii*	1	1/0	0.03	–	–	100
Genogroup 21	1	0/1	≤ 0.015	–	–	100
Genogroup 19	1	0/1	≤ 0.015	–	–	100
Genogroup 3	1	1/0	0.03	–	–	100
According to NCF/CF origin						
NCF	163	-	≤ 0.015 to 2	≤ 0.015	0.5	100
CF	67	-	≤ 0.015 to 16	0.03	1	97
According to non-susceptibility to TZP, MEM and SXT^***^						
TZP non-susceptible	23	10/13	≤ 0.015 to 16	0.125	2	91.3
MEM non-susceptible	27	12/15	≤ 0.015 to 16	0.125	2	96.3
SXT non-susceptible	35	14/21	≤ 0.015 to 16	0.06	1	94.3
TZP + MEM + SXT non-susceptible	8	1/7	0.06 to 16	0.5	16	87.5

CF: strain(s) from patient(s) with cystic fibrosis; I: susceptible, increased exposure; MEM: meropenem; MIC: minimal inhibitory concentration; n: number of strains; NCF: strain(s) from other patient(s) not suffering from cystic fibrosis; PK/PD: pharmacokinetics and pharmacodynamics; TZP: piperacillin-tazobactam; R: resistant; S: susceptible, standard dosing regimen; SXT: trimethoprim-sulfamethoxazole

^*^MIC_50_ and MIC_90_ were determined when the strain number exceeded three isolates

^**^EUCAST non-species PK/PD breakpoint for cefiderocol: S ≤ 2 mg/L; R > 2 mg/L

^***^In the absence of specific breakpoints for all species of *Achromobacter* genus, the inhibition zone diameter breakpoints for *A. xylosoxidans* had been applied to all *Achromobacter* species (EUCAST 2023, version 13.1): TZP (S ≥ 26 mm; R < 26 mm); MEM (S ≥ 26 mm; 20 mm ≤ I < 26 mm; R < 20 mm); SXT (S ≥ 26 mm; R < 26 mm)

### Susceptibility to cefiderocol within the collection of *Achromobacter* sp. strains studied

**Fig. 1:**
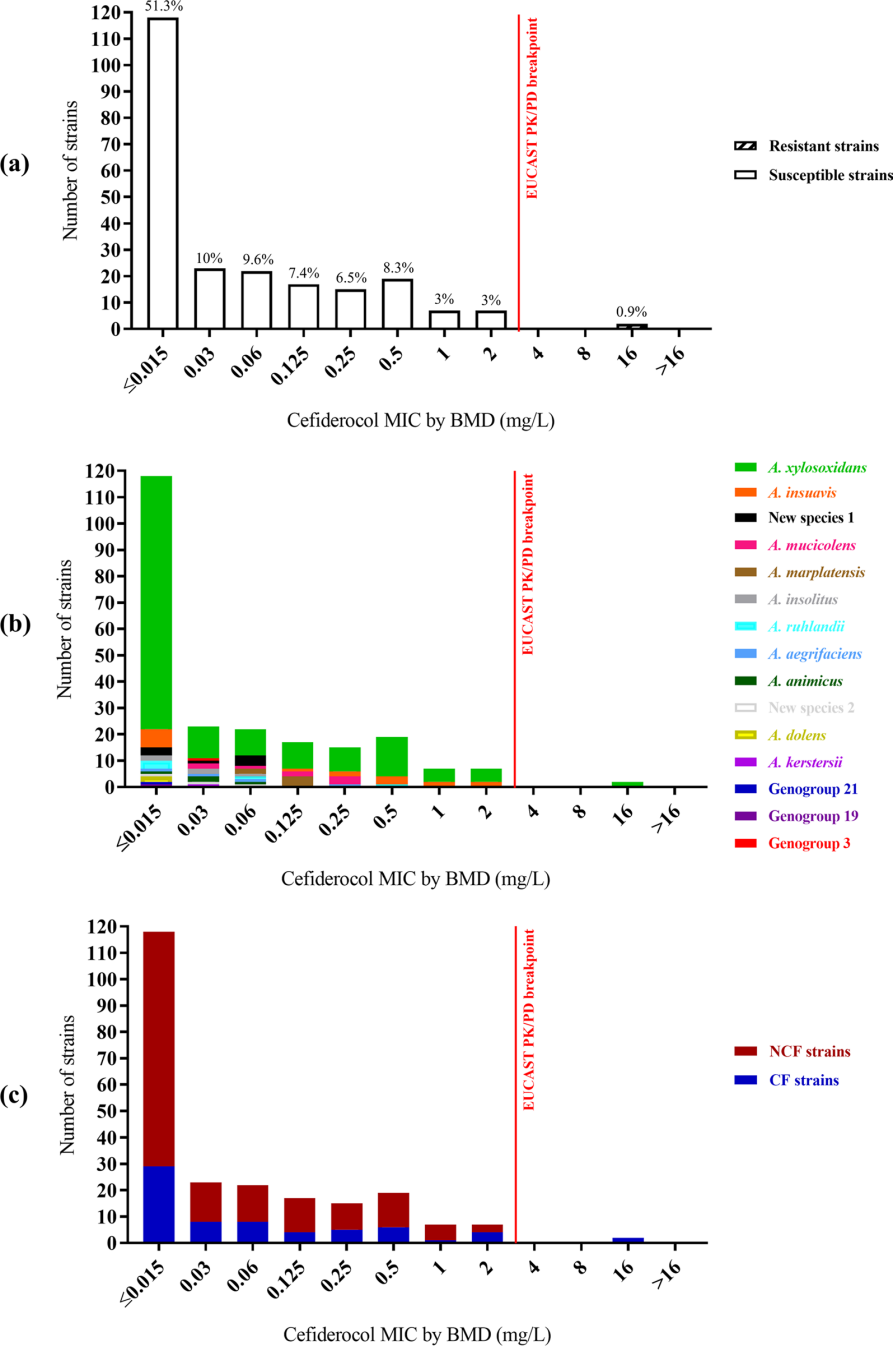
Distribution of cefiderocol MICs (mg/L) for the 230 *Achromobacter* strains of the study. MICs were determined by the BMD reference method and are presented for the overall 230 *Achromobacter* strains (**a**), according to *Achromobacter* species (**b**), and according to strains’ origin (NCF and CF) (**c**). EUCAST 2023 non-species PK/PD breakpoint for cefiderocol: S ≤ 2 mg/L; R > 2 mg/L. BMD: broth microdilution; CF: strains from patients with cystic fibrosis; EUCAST: European committee on antimicrobial susceptibility testing; MIC: minimal inhibitory concentration; NCF: strains from other patients not suffering from cystic fibrosis; PK/PD: pharmacokinetics and pharmacodynamics

Whatever the species and strain origin, MIC values ranged from ≤ 0.015 to 16 mg/L, with a MIC_50_ of ≤ 0.015 mg/L and a MIC_90_ of 0.5 mg/L. The large majority of *Achromobacter* sp. strains were susceptible to cefiderocol (99.1%, n = 228) and 0.9% (n = 2) of isolates displayed a MIC > 2 mg/L, above the EUCAST 2023 PK/PD breakpoint not related to a species, with MIC of 16 mg/L (Fig. 1a, Table 2).

### Susceptibility to cefiderocol according to *Achromobacter* species

All the 15 species were represented among the 228 susceptible strains (MICs ≤ 2 mg/L) whereas the two resistant strains (MICs > 2 mg/L) belonged to the species *A. xylosoxidans*, the most represented in our study (Fig. 1b, Table 2). No statistically significant relationship could be observed between the MICs of cefiderocol and the different species of the *Achromobacter* genus when these were considered individually (*p-value* = 0.19) (Additional file 2 a). Similar results were obtained when the analysis was limited to the 10 individual species with ≥ 3 strains (*p-value* = 0.10). However, the MICs of cefiderocol of the *A. xylosoxidans* species were statistically lower than that of other species when the analysis was limited to the seven individual species with ≥ 5strains (*p-value* = 0.03) or when species other than *A. xylosoxidans* were considered as a whole (*p-value* = 0.01) (Additional file 2 b).

### Susceptibility to cefiderocol according to strain origin

A total of 97% of CF strains (n = 65) and 100% of NCF strains (n = 163) were susceptible to cefiderocol (MIC ≤ 2 mg/L). More precisely, according to CF group, MIC values ranged from ≤ 0.015 to 16 mg/L, with a MIC_50_ of 0.03 mg/L and a MIC_90_ of 1 mg/L whereas, for the NCF group, MIC values ranged from ≤ 0.015 to 2 mg/L, with a MIC_50_ of ≤ 0.015 mg/L and a MIC_90_ of 0.5 mg/L. The two resistant strains (MIC > 2 mg/L) were all of CF origin, suggesting that CF strains could be the least susceptible to cefiderocol (Fig. 1c, Table 2). However, no statistically significant relationship could be observed between the MICs of cefiderocol and the CF or NCF origin of the strains (*p-value* = 0.11) (Additional file 2 c).

### Susceptibility to cefiderocol according to TZP, MEM and SXT resistance profiles

All the 176 strains simultaneously susceptible to TZP, MEM (susceptible, standard dosing regimen and susceptible, increased exposure) and SXT were also susceptible to cefiderocol. None of the strains had isolated resistance to cefiderocol. Among the two strains resistant to cefiderocol, one was MDR strain (simultaneously resistant to TZP, MEM and SXT) and the other was resistant to both TZP and SXT but susceptible, increased exposure to MEM (Additional file 1).

Among the 27 MEM non-susceptible isolates of the study (11.7%), including 25 *A. xylosoxidans* and two *A. insuavis*, 26 remained susceptible to cefiderocol (range: ≤ 0.015 to 16 mg/L, MIC_50_: 0.125 mg/L, MIC_90_: 2 mg/L). Among the eight MDR strains of the study (3.5%), including seven *A. xylosoxidans* and one *A. insuavis*, seven remained susceptible to cefiderocol (range: 0.06 to 16 mg/L, MIC_50_: 0.5 mg/L, MIC_90_: 16 mg/L) (Table 2).

## Discussion

To the best of our knowledge, this study is the only one to have tested the susceptibility of cefiderocol using the BMD reference method on such a large panel of *Achromobacter* spp. strains accurately identified by *nrdA* gene sequencing and providing a full comparison to TZP, MEM and SXT susceptibility data. To date, only eight studies [32–39] have focused on determining susceptibility to cefiderocol of a series of *Achromobacter* spp., and only four of them [34–36, 38] used the BMD reference method (ID-CAMHB) (Table 3). Compared to the present study of 230 isolates, most of published articles have presented a limited number of strains ranging from one [37] to 74 strains [39], except for Takemura et al*.*, who recently studied a larger panel of 334 strains [36]. However, none of these articles used *nrdA* gene sequencing as the identification method except for Takemura et al*.* who performed whole-genome sequencing and MLST characterization on eight *Achromobacter* strains only (seven *A. xylosoxidans* and one *Achromobacter* sp., including six strains resistant to cefiderocol) [36]. When specified, the identification method was MALDI-TOF MS, suggesting low reliability of species identification and potentially explaining the low diversity of species identified (*A. xylosoxidans*, *A. insolitus*, *A. denitrificans*, *A. piechaudii* and *Achromobacter* sp.) compared to our study identifying 15 species among 230 strains. Moreover, only three studies specified the CF [32, 39] or NCF [35, 39] origin of the strains studied, and none compared cefiderocol susceptibility according to the origin of the strain.

**Table 3:** Cefiderocol susceptibility testing on *Achromobacter* spp. isolates in the literature and in this study

Ref	n	Origin	*Achromobacter* species	Identification method	Method(s) used for cefiderocol AST	IZD range (mm)	MIC range (mg/L)	MIC_50_ (mg/L)	MIC_90_ (mg/L)	Susceptibility (%)	Interpretation guidelines^*^
[32]	23	Sputum (CF patients)	*Achromobacter* sp.	MALDI-TOF MS (Bruker)	MIC with EUMDROXF® plate Sensititre (standard CAMHB)	–	≤ 0.03 to > 8	0.25	1	91	EUCAST
[33]	8	–	*A. xylosoxidans*	MALDI-TOF MS (Bruker)	MIC with Sensititre lyophilized BMD panel (standard CAMHB)	–	0.06 to 1	0.25	1	100	EUCAST
[34]	12	–	*A. xylosoxidans*	MALDI-TOF MS (NS brand)	MIC with BMD reference method (ID-CAMHB)	–	0.25 to 2	0.5	1	100	EUCAST, CLSI
[35]	15	Blood (> 90%) (cancer patients)	*Achromobacter* sp.	–	MIC with BMD reference method (ID-CAMHB)	–	–	–	0.125	100	CLSI
[36]	334	Respiratory tract (76.6%) Blood (7.2%) Skin (6%), gastrointestinal (4.8%) and urinary (3.9%) tract, unknown (1.5%)	*A. xylosoxidans* (93.1%) *A. insolitus* (3.3%), *Achromobacter* sp. (2.7%) *A. denitrificans* (0.6%) *A. piechaudii* (0.3%)	MALDI-TOF MS (Bruker) + WGS and MLST characterization for 8 strains (2.4%)	MIC with BMD reference method (ID-CAMHB)	–	≤ 0.03 to > 64	0.06	0.5	96.7	EUCAST
[37]	1	–	*A. xylosoxidans*	MALDI-TOF MS (bioMérieux)	MIC with Sensititre™ cefiderocol MIC panel CMP1SHIH (ID-CAMHB)	–	0.06	–	–	100	EUCAST
[38]	15	Blood (60%), respiratory tract (40%)	*A. xylosoxidans*	MALDI-TOF MS (Bruker)	MIC with BMD reference method (ID-CAMHB) + MIC with UMIC® BMD panel + disk diffusion method (Oxoid, Liofilchem, Mast Diagnostics)	28–36	≤ 0.03 to 0.25	≤ 0.03	0.25	100	EUCAST
[39]	74	Respiratory tract from people with CF (92%) and bronchiectasis (8%)	*A. xylosoxidans* (93.2%), *Achromobacter* sp. (6.8%)	MALDI-TOF MS (NS brand)	MIC with UMIC® BMD panel	–	–	0.5	8	87.8	EUCAST
[27] (case 1)	1	Blood/implantable device (NCF patient)	*A. xylosoxidans*	MALDI-TOF MS (Bruker)	MIC with BMD reference method (ID-CAMHB) + disk diffusion method (Liofilchem)	35	1	–	–	100	EUCAST
[14, 24, 28] (case 2)	1	Blood/implantable device (NCF patient)	*A. xylosoxidans*	–	Disk diffusion method (NS brand)	21	–	–	–	–	–
[14, 26] (case 3)	2	Blood (CF patient)	*A. xylosoxidans*	–	MIC with BMD reference method (ID-CAMHB)	–	≤ 0.03 to 0.12	–	–	100	CLSI
[14, 26] (case 4)	2	Respiratory tract (CF patient)	*Achromobacter* sp.	–	MIC with BMD reference method (ID-CAMHB)	–	1 to 64	–	–	50	CLSI
[14, 26] (case 5)	1	Respiratory tract (CF patient)	*A. xylosoxidans*	–	MIC with BMD reference method (ID-CAMHB)	–	1	–	–	100	CLSI
[14, 26] (case 6)	1	Respiratory tract (CF patient)	*Achromobacter* sp.	–	Disk diffusion method (NS brand)	20	–	–	–	–	CLSI
[14, 26] (case 7)	2	Respiratory tract (CF patient)	*A. denitrificans*	–	MIC with BMD reference method (ID-CAMHB) + disk diffusion method (NS brand)	Strain 2: 17	Strain 1: 0.06	–	–	Strain 1: 100	Strain 1: CLSI
[14, 22, 26, 29] (case 8)	3	Respiratory tract (CF patient)	*Achromobacter* sp. *A. xylosoxidans* *A. ruhlandii*	–	MIC with BMD reference method (ID-CAMHB)	–	1 to 32	16	32	33.3	CLSI
[26] (case 9)	1	Respiratory tract (CF patient)	*Achromobacter* sp.	–	MIC with BMD reference method (ID-CAMHB)	–	> 64	–	–	0	CLSI
[26] (case 10)	1	Respiratory tract (CF patient)	*Achromobacter* sp.	–	MIC with BMD reference method (ID-CAMHB)	–	0.06	–	–	100	CLSI
This study	230	see Table 1 (mainly respiratory tract from both NCF and CF patients)	see Table 2 (mainly *A. xylosoxidans* and *A. insuavis*)	*nrdA* gene-based phylogeny	MIC with BMD reference method (ID-CAMHB)	–	≤ 0.015 to 16	≤ 0.015	0.5	99.1	EUCAST

AST: antimicrobial susceptibility testing; BMD: broth microdilution; CF: strain(s) from patient(s) with cystic fibrosis; CLSI: clinical & laboratory standards institute; EUCAST: European committee on antimicrobial susceptibility testing; (ID)-CAMHB: (iron-depleted) cation-adjusted Mueller–Hinton broth; IZD: inhibition zone diameter; MALDI-TOF MS: matrix-assisted laser desorption/ionization-time of flight mass spectrometry; MIC: minimal inhibitory concentration; MLST: multilocus sequence type testing; n: number of isolates; NCF: strain(s) from other patient(s) not suffering from cystic fibrosis; NS: not specified; Ref.: reference; PK/PD: pharmacokinetics and pharmacodynamics; WGS: whole-genome sequencing

^*^EUCAST 2023 non-species PK/PD breakpoint for cefiderocol: S ≤ 2 mg/L; R > 2 mg/L. The Investigational CLSI MIC breakpoints for the “other non-Enterobacterales” category were used with values of S ≤ 4 mg/L and R ≥ 16 mg/L

Among the overall 482 *Achromobacter* spp. strains included in these eight studies, a large majority of strains were susceptible to cefiderocol, with MIC_50_ values ranging from ≤ 0.03 mg/L [38] to 0.5 mg/L [34, 39] and MIC_90_ values ranging from 0.125 mg/L [35] to 1 mg/L except for the study of Tunney et al. who reported a MIC_90_ of 8 mg/L [39]. Indeed, in the latter study, nine strains were found resistant to cefiderocol out of the 74 beyond investigation using the Bruker UMIC cefiderocol assay (Bruker Daltonics GmbH and Co. KG), resulting in an exceptionally high rate of resistance (12.2%) compared to other studies [39]. Taking all these studies together, 22 strains (4.6%) were resistant to cefiderocol: two strains (one *A. xylosoxidans* and one *A. insolitus*) from sputum of CF patients with MICs ≥ 8 mg/L with EUMDROXF® plate Sensititre (standard CAMHB) [32], six strains (five *A. xylosoxidans* and one *Achromobacter* sp.) with MICs ≥ 16 mg/L with BMD reference method (origin not specified) and 14 other strains with no available associated information on species or origin [36, 39]. Among these 22 strains resistant to cefiderocol, at least seven isolates (≥ 31.8%) were non-susceptible to MEM (no data given on both TZP and SXT susceptibilities).

The limitations of our study include the small number of both carbapenem non-susceptible strains (27 isolates) and MDR strains (eight isolates), isolates for which cefiderocol may be necessary in routine clinical practice.

In vitro susceptibility data on cefiderocol are crucial, especially as an increasing number of patients infected with *Achromobacter* spp. are being treated with this antibiotic. To date, the literature reports 10 cases of serious *Achromobacter* spp. infections treated with cefiderocol (always in combination with other antibiotics ± bacteriophage), and showed occasional data on AST of cefiderocol (Table 3) [14, 20, 22–29]. Among these 10 cases, eight were CF patients including five with lung transplant, and two were NCF patients. Most patients had *Achromobacter* sp. infections of the respiratory tract (7/10) including one with empyema, or less frequently, bacteremia (3/10) including one with endocarditis. In total, five cases of infection were reported with *A. xylosoxidans*, one with *A. denitrificans*, one with *A. ruhlandii* and others with non-specified *Achromobacter* species, even though none of these studies used *nrdA* gene sequencing as the identification method. Isolates from three patients (cases 4, 8 and 9) exhibited cefiderocol-resistance (MIC of 64 mg/L, MIC of 32 mg/L and MIC > 64 mg/L, respectively) despite the patients had not been treated with cefiderocol, as also observed in the present study. However, despite in vitro resistance of *Achromobacter* sp. to cefiderocol, two of these three patients reported clinical improvement when cefiderocol was associated with TZP plus colistin (case 4), or with MEM-vaborbactam plus specific bacteriophage Ax2CJ45Φ2 (case 8). The third remaining patient (case 9, a post-lung transplant CF patient) remained stable with no clinical improvement, despite the combination of cefiderocol with both ceftazidime–avibactam and SXT [26] (Table 3).

In vitro resistance to cefiderocol is not synonymous with clinical failure, as cefiderocol is commonly used in combination with other antimicrobials. Further in vivo experiments are thus necessary to better understand the potency of cefiderocol against these uncommon pathogens.

## Conclusion and outlooks

*Achromobacter* spp. strains are highly susceptible to cefiderocol, whatever their origin or species. Moreover, real-life data on the effectiveness of cefiderocol are promising, particularly for severely infected patients with carbapenem-resistant *Achromobacter* sp. [14, 36]. Cefiderocol can be considered as an additional promising option for salvage therapy of *Achromobacter* sp. infections even in difficult-to-treat cases.

Previous studies on cefiderocol susceptibility suggest that the development of cefiderocol resistance in non-fermenting pathogens like *A. baumannii* or *P. aeruginosa* requires various mechanisms, including mutations in iron transporters, defects in porin channels, and expression of specific β-lactamases [36, 40]. Thus, it is plausible that similar mechanisms may contribute to cefiderocol resistance in *Achromobacter* spp. It would now be interesting to study the mechanisms of cefiderocol resistance developed by the two strains with a cefiderocol MIC > 2 mg/L in the absence of cefiderocol exposure and, more generally, the evolution of cefiderocol resistance in the *Achromobacter* genus since its use, and to investigate its potential for the selection of resistance.

The BMD method is the reference method for in vitro susceptibility testing of cefiderocol but the preparation of ID-CAMHB is complex and time-consuming, making this technique difficult to apply routinely in a clinical microbiology laboratory [31]. Therefore, more widely accessible AST methods compared with the BMD reference method have been developed for cefiderocol susceptibility routine tests: disk diffusion method, cefiderocol-impregnated strips, both tested on regular MH-agar and several microdilution panels in liquid media [41]. It would now be interesting to compare the performance of commercially available tests with the BMD reference method to define the most accurate method for testing *Achromobacter* spp. This might be very helpful to the necessary ongoing monitoring of the susceptibility of *Achromobacter* spp. to cefiderocol, considering the increasing use of this newly available therapeutic option for managing infections caused by difficult-to-treat pathogens.

### Supplementary Information

Supplementary Material 1. Additional Table. *Achromobacter* spp. isolates of this study and results of antimicrobial susceptibility testing.

Supplementary Material 2. Additional Figure. Distribution of the cefiderocol MICs log (2) determined by the BMD reference method for the 230 *Achromobacter* strains of the study, according to species **(a)** and **(b)**, and according to origin (CF and NCF) **(c)**. The term “other” represents all species other than *A. xylosoxidans* **(b)**. Each strain is represented by a black dot and the average MIC is represented by a green triangle. CF, strains from patients with cystic fibrosis; NCF, strains from other patients not suffering from cystic fibrosis.

## Data Availability

Not applicable.

## Abbreviations

AST: Antimicrobial susceptibility testing
BMD: Broth microdilution
CF: Cystic fibrosis
EUCAST: European committee on antimicrobial susceptibility testing
GNB: Gram-negative bacilli
I: Susceptible, increased exposure
ID-CAMHB: Iron-depleted cation-adjusted Mueller–Hinton broth
IZD: Inhibition zone diameter
MALDI-TOF MS: Matrix-assisted laser desorption/ionization-time of flight mass spectrometry
MH: Mueller–Hinton
MDR: Multidrug resistant
MEM: Meropenem
MIC: Minimal inhibitory concentration
MLST: Multilocus sequence typing
NCF: Non-cystic fibrosis
PBP: Penicillin-binding proteins
PK/PD: Pharmacokinetics and pharmacodynamics
TZP: Piperacillin–tazobactam
R: Resistant
S: Susceptible, standard dosing regimen
SXT: Trimethoprim–sulfamethoxazole
