# Supplementary figures and images for: Cefiderocol susceptibility of Achromobacter spp.: study of an accurately identified collection of 230 strains
Source: Ann Clin Microbiol Antimicrob. 2024 Jun 17;23:54. doi: 10.1186/s12941-024-00709-z (PMC11184864; doi:10.1186/s12941-024-00709-z)

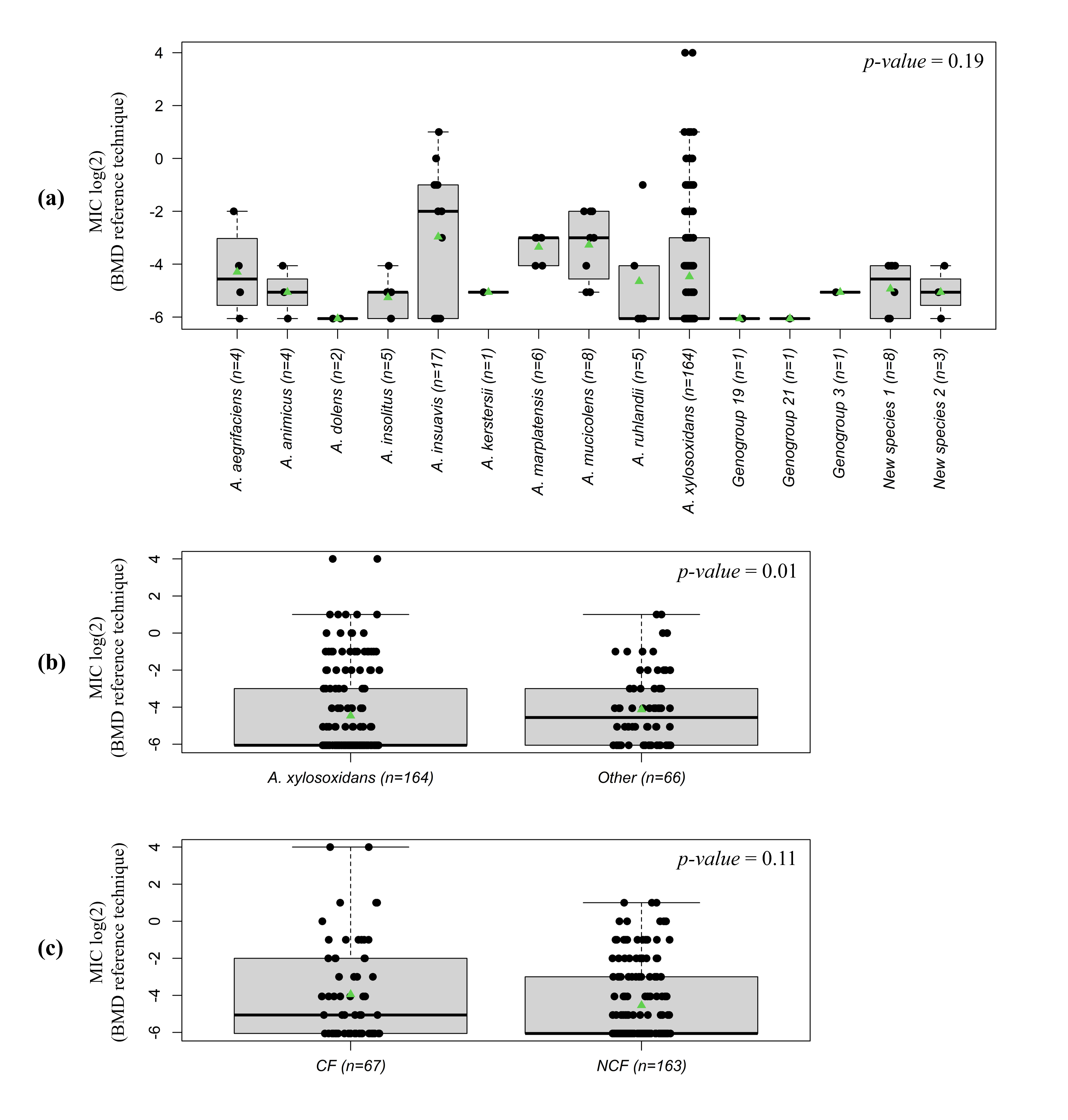

Supplement: Supplementary file 2 — Supplementary Material 2. Additional Figure. Distribution of the cefiderocol MICs log (2) determined by the BMD reference method for the 230 Achromobacter strains of the study, according to species (a) and (b), and according to origin (CF and NCF) (c). The term “other” represents all species other than A. xylosoxidans (b). Each strain is represented by a black dot and the average MIC is represented by a green triangle. CF, strains from patients with cystic fibrosis; NCF, strains from other patients not suffering from cystic fibrosis. [file 12941_2024_709_MOESM2_ESM.tif]
